# Development and enhancement of metamaterial-inspired Ag-GaAs THz MIMO antenna with optimized diversity metrics using data-driven machine learning algorithms for future 6G networks

**DOI:** 10.1038/s41598-025-28978-4

**Published:** 2025-12-29

**Authors:** Ammar Armghan, Vishalkumar Mandaliya, Meshari Alsharari, Khaled Aliqab, Slim Ben Chaabane, Aymen Flah

**Affiliations:** 1 Department of Electrical Engineering, College of Engineering, Jouf University, Sakaka, 72388 Saudi Arabia; 2 Tech Mahindra Americas, 5700 Democracy Dr Ste 2000, Plano, TX 75024 USA; 3 Computer Engineering Department, Faculty of Computers and Information Technology, University of Tabuk, Tabuk, 47512 Saudi Arabia; 4 Applied Science Research Center, Applied Science Private University, Amman, 11931 Jordan; 5 ENET Centre, CEET, VSB-Technical University of Ostrava, Ostrava, Czech Republic; 6 College of Engineering, University of Business and Technology (UBT), Jeddah, 21448 Saudi Arabia

**Keywords:** MIMO antenna, 6G networks, Metamaterial, Tuning, Machine learning, Optimization, Engineering, Mathematics and computing

## Abstract

The MIMO antenna design is specifically engineered to support optimized performance in emerging 6G networks. Utilizing advanced techniques such as metamaterials and machine learning algorithms, the antenna system achieves high data rates, improved diversity, and robust signal reliability, making it ideal for next-generation ultra-fast and intelligent wireless communication technologies. Our advanced metamaterial configuration demonstrates high gain and bandwidth. A low ECC value 0.0004 shows minimal correlation, ensuring better signal diversity and improved system performance. Similarly, a high diversity gain confirms the antenna’s efficiency in maintaining robust signal reception under varying conditions. The CCL values of 0.0916 bits/Hz bits/Hz provide insight into the information-carrying capacity of the MIMO configuration. The MIMO antenna design achieves a maximum gain of 8.9 dBi and a wide bandwidth of 30 THz. This performance is attained through a combination of parametric optimization and machine learning techniques, enhancing both efficiency and operational range. The machine learning algorithms used for optimization yield a high R² value of 0.99, indicating excellent prediction accuracy. The proposed antenna, featuring metamaterial characteristics, demonstrates strong potential for next-generation 6G networks, offering enhanced performance, efficiency, and compact design integration.

## Introduction

MIMO antennas are used widely nowadays because of their exceptional capability to support high-speed networks and improved spectral efficiency. Unlike conventional antenna designs, MIMO technology utilizes multiple transmitting and receiving antennas, which enables simultaneous data transmission over multiple spatial channels^1^. This spatial multiplexing significantly enhances the throughput and reliability of communication networks, particularly in environments with high user density and signal interference^2^. With higher data requirements, MIMO antennas are increasingly adopted across various platforms, including smartphones, base stations, etc^3^. The ability to improve parameters makes them highly suitable for high-speed communication^4^. A compact coradiator annular ring MIMO antenna with orthogonal polarization offers great bandwidth, high isolation, and 8 dBi gain. The structure is also fabricated and tested for the IoT and 6G communication applications^5^.

The emergence of 6G communication networks demands antenna systems capable of supporting higher data speeds^6^. To meet these ambitious requirements, MIMO antennas must be optimized for high gain and wide bandwidth performance^7^. High gain ensures strong and focused signal transmission over longer distances, while wide bandwidth allows for the accommodation of vast amounts of data across multiple channels. These two parameters are crucial for enabling real-time communication, immersive technologies, and intelligent systems envisioned in 6G frameworks^8^. Efficient MIMO antenna design must also consider compact size, energy efficiency, and the ability to mitigate interference and multipath fading. Integrating metamaterials and tunable components such as graphene gives changing channel conditions^9^. Therefore, achieving high gain and bandwidth through innovative and intelligent MIMO antenna designs is fundamental for 6G networks^10^. A MIMO antenna with SIW Cavity is applied for advanced 5G and 6G wireless communication systems. This innovative antenna supports four distinct frequency bands within a compact form factor, offering high isolation and excellent MIMO performance. The shared-aperture design enables efficient space utilization, while the dual-mode SIW cavity enhances gain, bandwidth, and polarization diversity—making it highly suitable for future high-speed, multi-band wireless networks^11,12^.

One of the key strategies for enhancing MIMO antenna performance is the integration of metamaterials into the antenna structure. Metamaterials possess engineered electromagnetic properties with artificial magnetism, which give better antenna performance^13^. By incorporating metamaterials, critical parameters like isolation, gain can be significantly improved. Enhanced gain ensures stronger signal transmission and reception, while broader bandwidth supports the high data rates and low latency required by modern wireless systems, especially in 6G communication^14^. Furthermore, metamaterials enable antenna design applicable in mobile and space-constrained devices. Their unique capability to manipulate electromagnetic waves leads to reduced signal loss, better beam steering, and improved diversity performance. As a result, the use of metamaterials is a powerful approach to overcoming traditional limitations and optimizing MIMO antenna designs for advanced wireless communication applications^15^.

Metamaterial-based MIMO antennas are increasingly employed to meaningfully enhance the performance. These advanced antenna structures leverage to manipulate wave propagation in ways not possible with conventional materials^16^. One of the most effective methods of integrating metamaterials into MIMO antenna design is through the incorporation of SRR and thin wire arrays into the radiating patch. These inclusions enable improved control over current distribution and electromagnetic coupling, leading to increased gain, bandwidth, and isolation between MIMO elements^17^. Additionally, the use of defected ground structures (DGS) further contributes to performance optimization by suppressing surface waves, reducing mutual coupling, and enhancing impedance matching. Such designs offer a compact and efficient solution for next-generation applications^18^. Overall, metamaterial-enhanced MIMO antennas are promising candidates for 6G high-speed networks^19^.

The advent of 6G communication demands antennas that are not only compact but also exhibit exceptional efficiency with a wide bandwidth to get the massive data transmission^20^. In this context, metamaterial-based MIMO antennas are giving higher performance. These antennas incorporate engineered structures such as negative index materials, split ring resonators, and artificial magnetic conductors, enabling improved control over wave propagation, reduced mutual coupling, and enhanced signal integrity^21^. By integrating metamaterials into MIMO configurations, it becomes possible to significantly boost system capacity, diversity gain, and spectral efficiency—crucial factors for the high-speed and ultra-reliable performance required in 6G networks. Additionally, these designs support miniaturization without compromising performance, which is essential for embedding antennas into increasingly compact devices. Thus, metamaterial MIMO antennas represent a vital technological advancement for fulfilling the stringent requirements of emerging 6G communication systems^22^.

The increasing requirements for 6G communication need a wide bandwidth but also achieve high gain for enhanced signal strength and reliability. To further improve performance, machine learning is used alongside parametric optimization methods. These approaches enable precise tuning of critical design parameters, resulting in optimal electromagnetic behavior and improved overall efficiency. The integration of intelligent optimization ensures the antenna meets the stringent need for 5G, 6G, and beyond, where speed and performance are crucial. The proposed metamaterial-based MIMO antenna exhibits remarkable enhancements in both gain and bandwidth, supported by strong performance indicators. The exceptionally low envelope correlation coefficient (ECC) of 0.0004 signifies excellent isolation and superior signal diversity, ensuring reliable communication performance. Furthermore, the high diversity gain highlights the antenna’s capability to sustain efficient signal reception under diverse propagation conditions. The calculated channel capacity loss (CCL) of 0.0916 bits/Hz indicates the system’s strong information-handling efficiency. Overall, the optimized design achieves an impressive peak gain of 8.9 dBi and an extensive operational bandwidth of 30 THz, confirming its suitability for next-generation high-speed wireless communication systems.

The novelty of the presented design is given below:

- The MIMO antenna is optimized specifically for emerging 6G wireless communication systems.
- Integration of metamaterials and machine learning enhances electromagnetic and predictive performance.
- Machine learning optimization achieves a high accuracy with an R² value of 0.99.
- The design attains a maximum gain of 8.9 dBi and an ultra-wide bandwidth of 30 THz.
- A low ECC of 0.0004 ensures excellent signal diversity and minimal correlation.
- High diversity gain confirms stable and robust signal reception.
- The CCL value of 0.0916 bits/Hz demonstrates strong data transmission capability.
- The compact metamaterial structure offers enhanced efficiency and suitability for next-generation 6G integration.

## Design and modelling

**Fig. 1:**
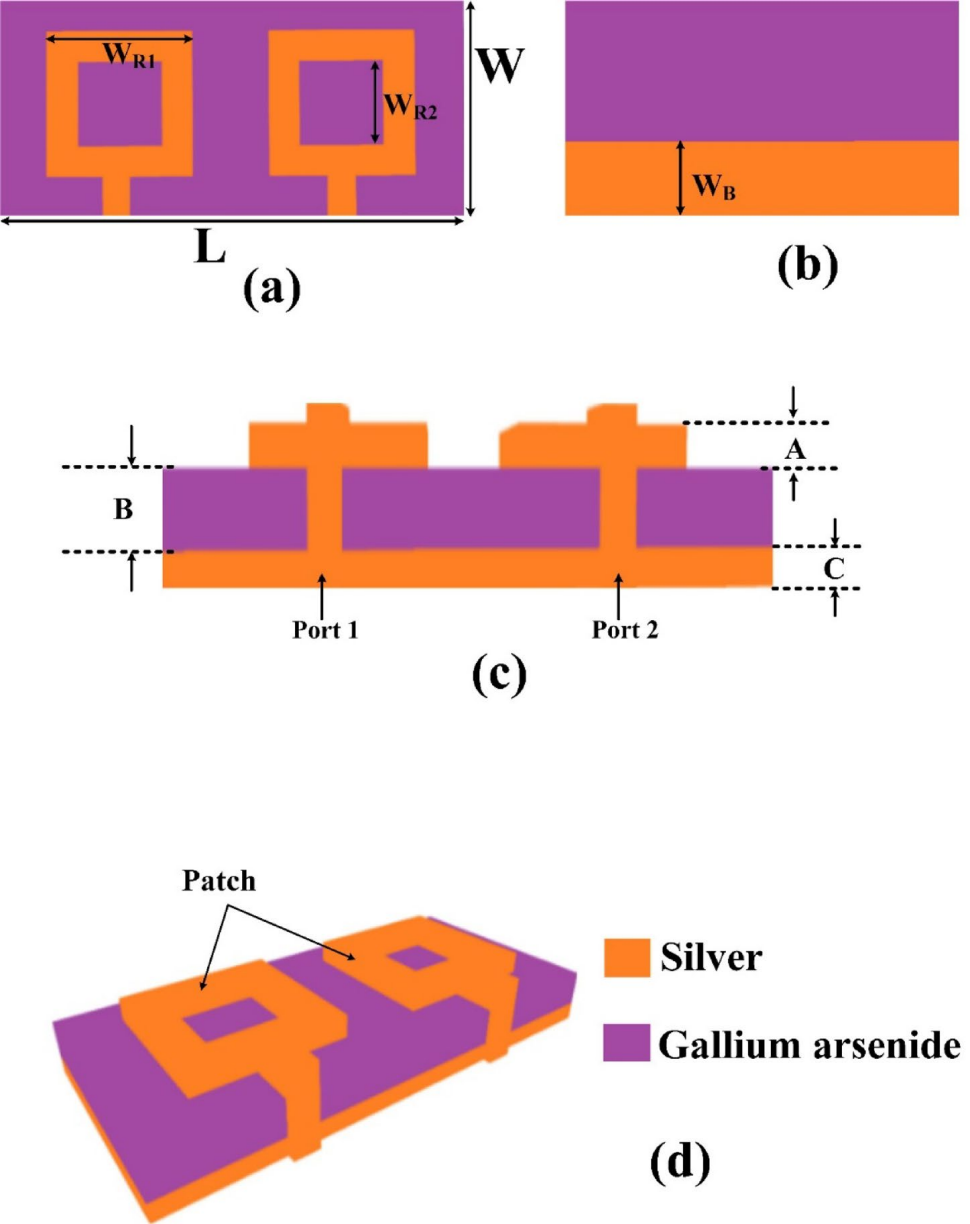
MIMO antenna design. **(a-d)** Different views of the designed structure. The structure measures 110 μm in length and 55 μm in width, ensuring compactness for microelectronic or nanoscale applications.

A detailed examination of the proposed antenna design, focusing on the implementation of metamaterial features to enhance its performance is presented. Specifically, an O-shaped metamaterial structure is incorporated in the top layer, while the ground plane is intentionally modified using a defected ground structure (DGS) to achieve better control over the electromagnetic characteristics. This structural modification gives a bandwidth, gain, and isolation. A two-port MIMO configuration is employed in this research to ensure high efficiency while maintaining a compact form factor. This configuration allows for enhanced diversity performance and reduced mutual coupling, which are essential in modern high-speed communication systems. The incorporation of the DGS further contributes to improved impedance matching and reduced surface wave propagation. Figure 1 illustrates various views of the proposed antenna layout, providing a clear visualization and the defected ground. The design with a width of 35 μm, strategically chosen to optimize performance. The substrate is 110 μm in length and 55 μm in width, making it good for a small size.

In the proposed antenna design, a metamaterial configuration is integrated into a square patch structure to enhance its electromagnetic performance^23^. The original square patch has an area of 35 μm², serving as the primary radiating element. To incorporate the metamaterial effect, a 20 μm² section is strategically removed from the patch, forming a distinct O-shaped geometry. This deliberate subtraction not only alters the surface current distribution but also introduces the desired metamaterial behavior within the radiating structure. The O-shape gives the tuning and improves efficiency.

To characterize a metamaterial, its effective electromagnetic properties are extracted using standard retrieval methods. These give scattering parameters obtained from full-wave simulations. Specifically, the S-parameters are used in Eqs. (1–5). This gives the metamaterial O-shape influences the antenna’s performance, providing valuable insight into wave propagation, impedance behavior, and the interaction of electromagnetic fields with the modified structure^24,25^.

1 $$\:z=\:\pm\:\sqrt{\frac{{(1+{S}_{11})}^{2}-{{S}_{21}}^{2}}{{(1-{S}_{11})}^{2}-{{S}_{21}}^{2}}}$$

2 $$\:{e}^{in{k}_{0}d}=\:\frac{{S}_{21}}{1-{S}_{11}\frac{z-1}{z+1}}$$

3 $$\:n=\frac{1}{{k}_{0}d}\left[\left\{{\left[\mathrm{ln}{(e}^{in{k}_{0}d})\right]}^{"}+2m\pi\:\right\}-i{\left[\mathrm{ln}{(e}^{in{k}_{0}d})\right]}^{{\prime\:}}\right]$$

4 $$\:\varepsilon\:=\frac{n}{z}$$

5 $$\:\mu\:=nz$$

### Fabrication approach

**Fig. 2:**
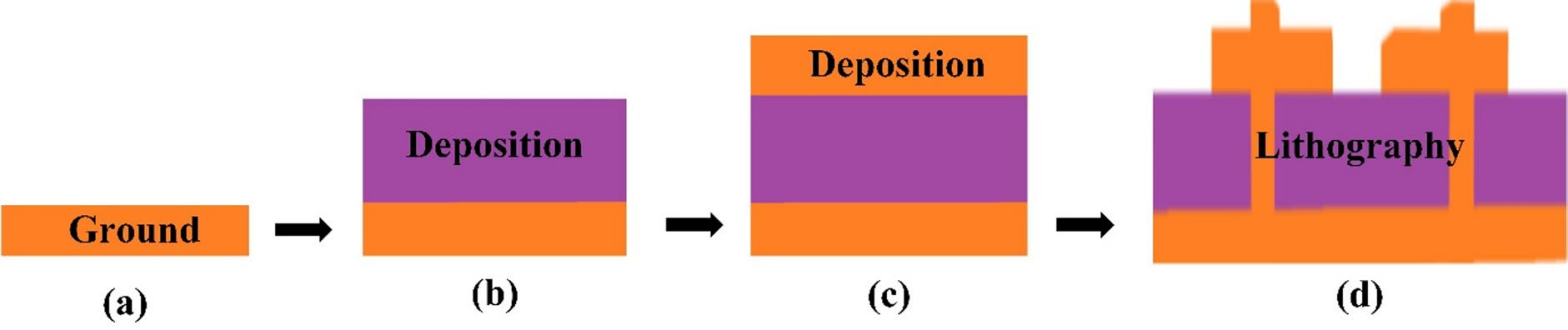
Fabrication approach for the layers including deposition and lithography.

The MIM structure is constructed through a sequential fabrication process involving the deposition and patterning of multiple layers and is presented in Fig. 2. Initially, a conductive ground layer is uniformly deposited onto the base platform, followed by the application of a dielectric substrate layer that acts as an insulating medium^26^. Subsequently, a metallic resonator layer is added on top to complete the layered configuration. After the deposition process, precision lithography techniques are employed to etch and define specific patterns in both the resonator and ground layers. Photolithography or electron-beam lithography may be used depending on the required resolution and application. These etching steps are crucial for forming the desired resonant geometry and enabling the structure to exhibit its targeted electromagnetic or optical characteristics. Careful alignment and control during the lithographic process ensure high structural accuracy and repeatability. This multilayered fabrication approach enables the realization of high-performance MIM structures suitable for applications in sensing, filtering, and photonic or plasmonic device engineering.

## Results analysis

**Fig. 3:**
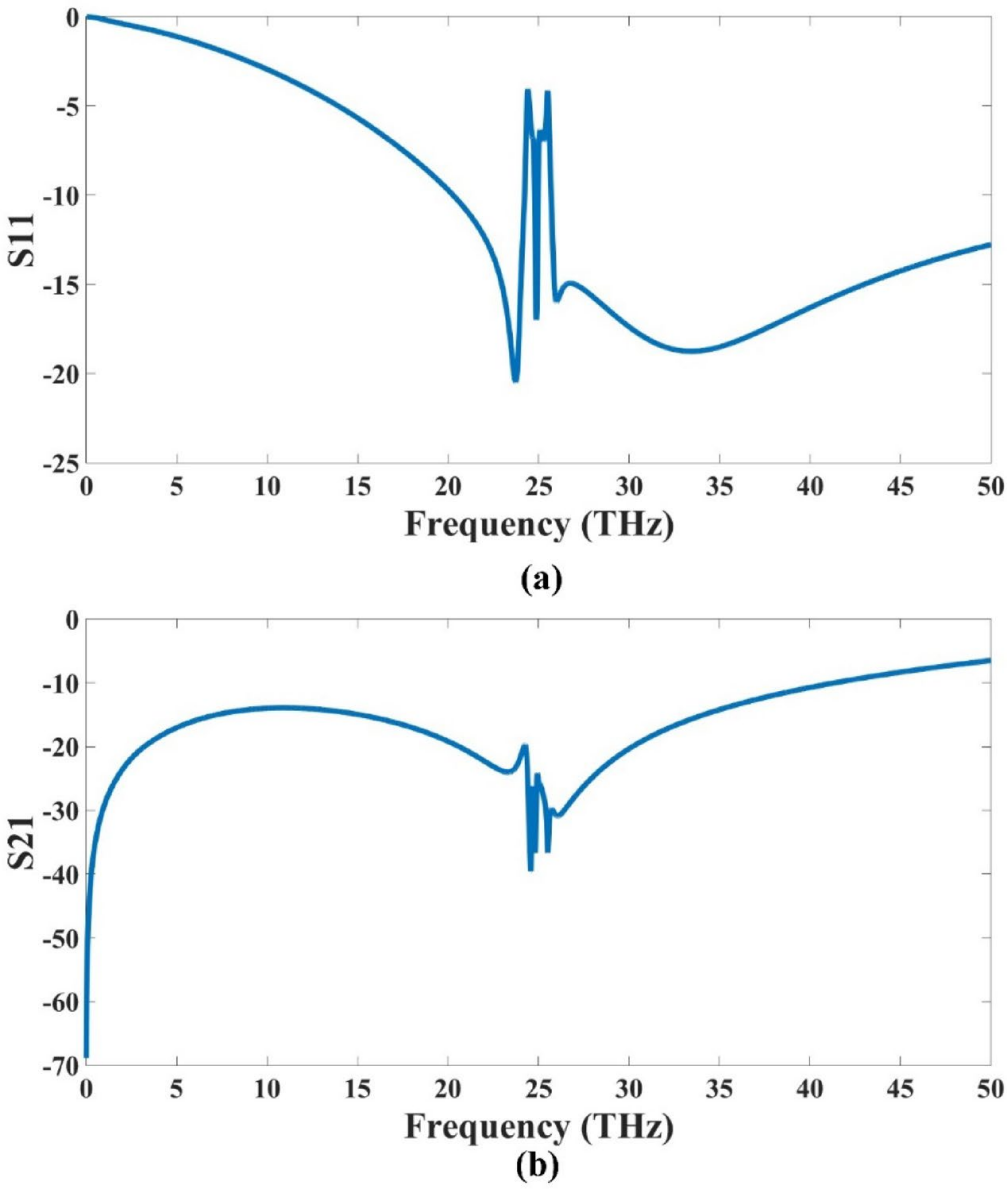
S-parameter results **(a)** S11 **(b)** S21. The highest bandwidth achieved is 30 THz (with the first band 5.5 (18.5–24) THz and the second band is 24.5 (25.5–50) THz).

The proposed design illustrated in Fig. 1 is thoroughly analyzed using the HFSS, a full-wave electromagnetic simulation tool widely used for antenna and RF structure modeling. Following the simulation process, the obtained results are depicted in Fig. 3, which presents the S-parameter characteristics of the MIMO antenna configuration. The results specifically highlight the performance in terms of S_11_ and transmission coefficient (S21), which are critical parameters for evaluating antenna efficiency and isolation. The S_11_ values indicate strong impedance matching and low reflection at the input ports, suggesting efficient radiation and minimal power loss. Meanwhile, the S_21_ results demonstrate effective isolation between antenna elements, an essential criterion for optimal MIMO performance. Overall, the simulated data confirms that the antenna design exhibits desirable electromagnetic performance in 6G communication.

The results illustrated in Fig. 3 validate the presence of two distinct operating frequency bands in the proposed design. The broader bandwidth spans around 30 THz. This dual-band behavior indicates that the structure is capable of supporting wide-frequency applications efficiently. Furthermore, the transmission coefficient (S21) results highlight strong isolation characteristics between the ports, which is a crucial requirement for MIMO systems. The antenna is enhancing overall system performance, reducing interference, and improving signal integrity. The wide bandwidths observed are highly suitable for high-speed, broadband communication technologies. Additionally, the sharp transitions and distinct separation between the frequency bands further affirm the effectiveness and precision of the resonator structure used. Overall, the S-parameter analysis confirms that the proposed design successfully achieves dual-band operation with excellent port isolation, making it ideal for next-generation terahertz communication and sensing systems.

**Fig. 4:**
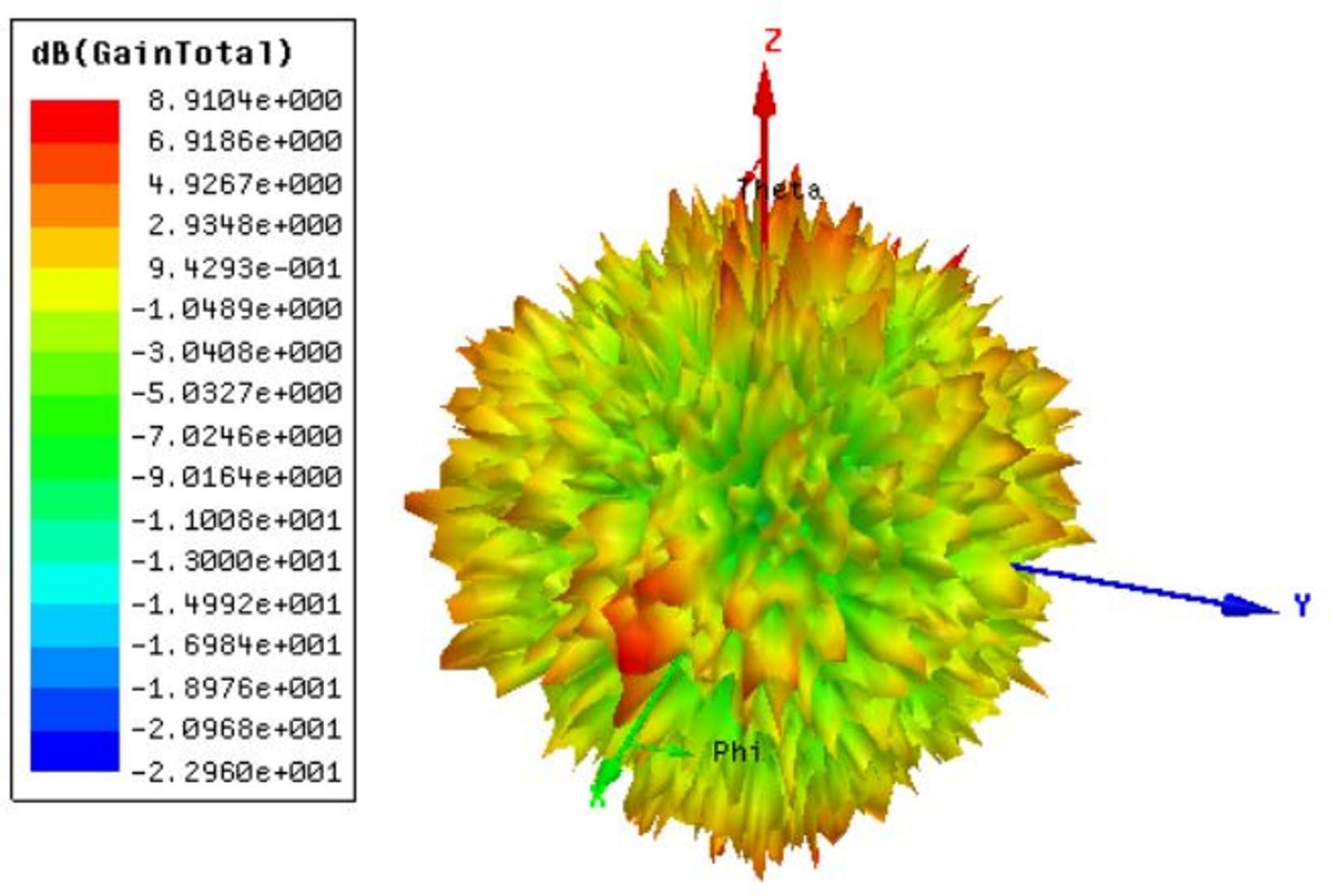
Gain polar plot results for the o-shaped metamaterial design. The highest gain of 8.91 dBi.

The gain response is given using the gain polar plot, as depicted in Fig. 4. The analysis reveals that the antenna achieves a peak gain of 8.91 dBi, which is considered highly effective, especially in the context of wideband communication systems. This significant gain value complements the broad bandwidth characteristics observed in Fig. 3, where the design demonstrated 30 THz bandwidths (with the first band 5.5 (18.5–24) THz and the second band is 24.5 (25.5–50) THz). The strong gain performance not only supports efficient radiation over a wide frequency range but also enhances signal strength and reliability, which are crucial for high-speed communication. The symmetrical nature of the gain response shows a better radiation pattern. Overall, the combination of high gain and wide bandwidth confirms the suitability of the structure for advanced terahertz MIMO systems, ensuring both high isolation and robust electromagnetic performance in demanding wireless environments.

### MIMO design optimization

**Fig. 5:**
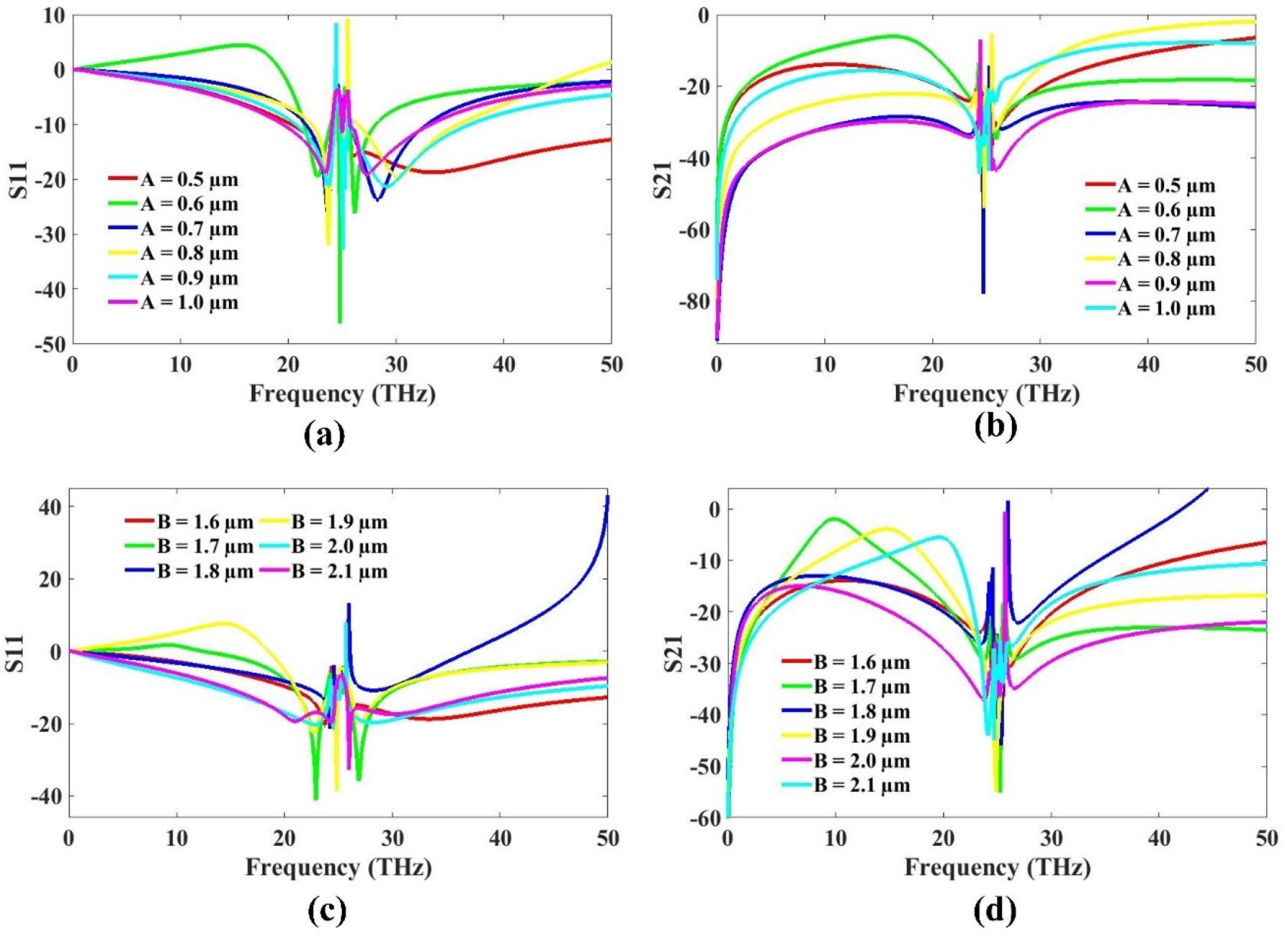
MIMO optimization analysis for patch and substrate height **(a)** S11 **(b)** S21 for patch height **(c)** S11 and **(d)** S21 for substrate height.

**Fig. 6:**
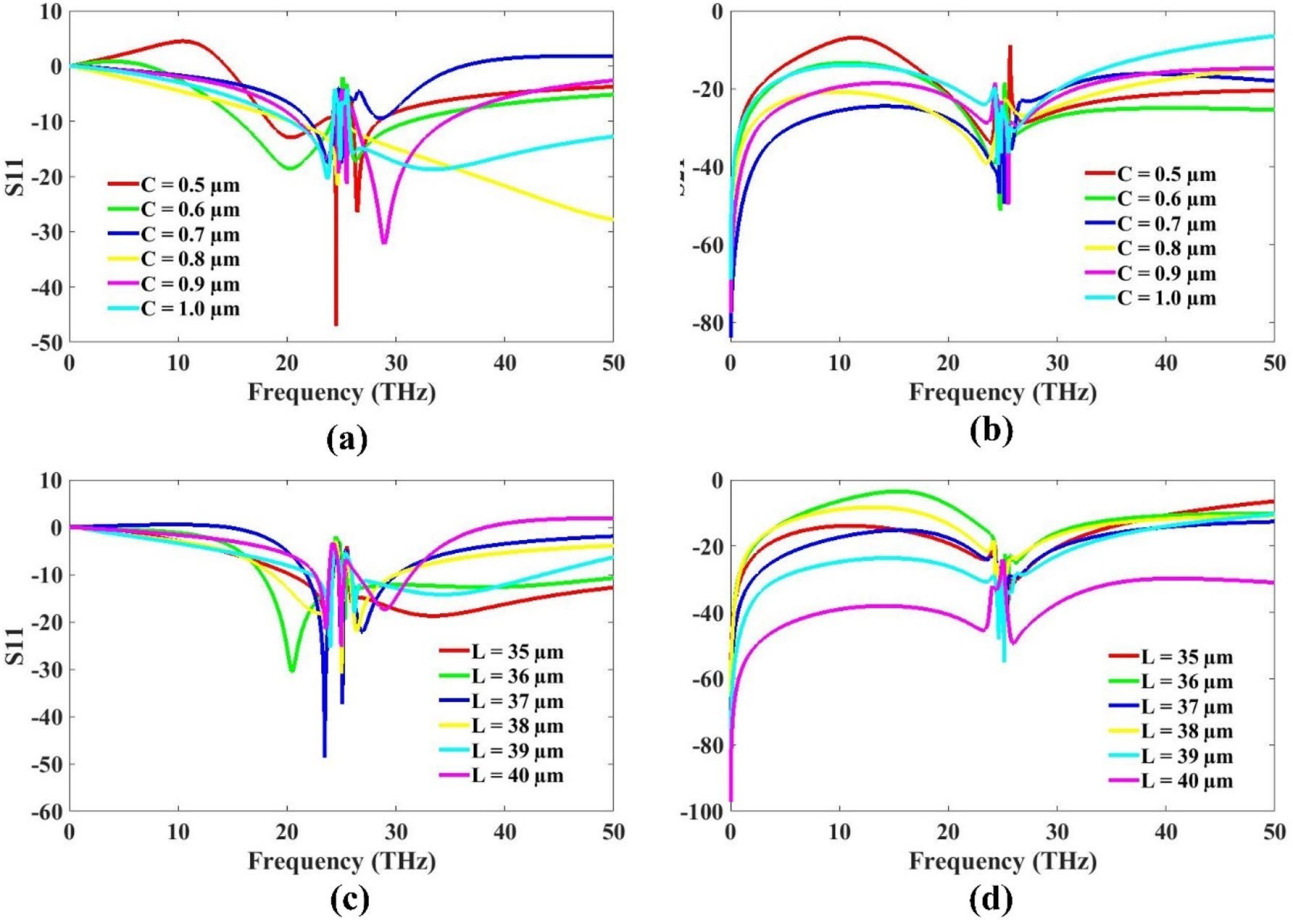
MIMO optimization analysis for ground plane height and patch length **(a)** S11 **(b)** S21 for ground plane height **(c)** S11 and **(d)** S21 for patch length.

The proposed MIMO antenna design has undergone comprehensive parametric optimization to refine its structural and electrical parameters for optimal performance. By systematically varying key design dimensions and material properties, the optimization process aims to achieve the most effective configuration that delivers enhanced MIMO characteristics, such as improved gain, reduced mutual coupling, and wider bandwidth. This approach ensures that the antenna operates efficiently across its designated frequency bands while maintaining strong isolation and radiation stability. Advanced simulation tools were employed to perform iterative analysis and evaluate the influence of each parameter on overall antenna behavior. As a result, the most favorable parameter set was determined, leading to a highly optimized antenna structure. The response is given in Figs. 5 and 6, where notable improvements in performance metrics are evident. These results validate the effectiveness of the parametric tuning method in enhancing the functionality and reliability of the MIMO antenna.

In this study, key structural parameters such as the patch height and substrate height were systematically varied to analyze antenna results. The simulation results for these variations are presented in Fig. 5, showcasing the corresponding S11 and S21 responses. The patch height was adjusted within the range of 0.5 μm to 1 μm, while the substrate height was varied from 1.6 μm to 2 μm. These adjustments were aimed at identifying the optimal configuration that yields improved impedance matching and isolation between the antenna elements. Notably, the substrate was intentionally kept slightly thicker to retain the necessary capacitance for effective resonance behavior. The thickness of the substrate plays a critical role in achieving the desired electromagnetic properties, as it influences the field confinement and coupling between the layers. The observed S-parameter results clearly indicate the sensitivity of the antenna’s performance to these dimensional changes, highlighting the importance of precise geometric tuning in MIMO antenna optimization.

As evident from the simulation results, the lower values of both substrate height and patch height demonstrate superior performance in terms of S11 and S21 parameters. In the line plot shown in Fig. 5, these optimal results are clearly highlighted by the red-colored curve, which indicates better impedance matching and isolation characteristics. Specifically, a patch height of 0.5 μm and a substrate height of 1.6 μm were found to deliver the most favorable outcomes. These dimensions not only enhance the electrical performance of the antenna but also contribute to a more compact overall design. The reduced size resulting from these lower parameter values is advantageous in multiple aspects, particularly in minimizing fabrication costs and facilitating integration into compact systems. Additionally, a thinner substrate helps maintain the necessary capacitance for resonance without compromising structural stability. Hence, the final optimized design incorporates these lower values to achieve an effective balance between performance, size, and cost, making it well-suited for practical applications in advanced terahertz MIMO systems.

The influence of varying the ground plane height and patch length on the antenna performance is analyzed and the results are illustrated in Fig. 6. The ground plane height is adjusted between 0.5 μm and 1 μm, while the patch length is varied from 35 μm to 40 μm. Based on the simulation results, it is observed that a higher ground plane height leads to improved reflectance characteristics, which is beneficial for efficient antenna operation. Therefore, the optimized ground plane height is selected to be 1 μm. On the other hand, variations in patch length show that a smaller patch length contributes to a more compact antenna design without compromising performance. The reflectance and transmission characteristics remain favorable at the lower length, making the antenna more suitable for miniaturized applications. As a result, a patch length of 35 μm is chosen as the optimized value. This combination of dimensions not only enhances the antenna’s electrical performance but also supports reduced physical size, which is advantageous for integration in compact systems and cost-effective production.

### Metamaterial properties

In this section, the metamaterial properties of the proposed antenna design are analyzed using the reflectance and transmittance results obtained from simulations. The O-shaped metamaterial structure exhibits a unique electromagnetic response that enhances negative-index behavior through its symmetric geometry and strong magnetic–electric coupling. Unlike conventional Split Ring Resonator (SRR) designs, which rely primarily on magnetic resonance generated by split gaps and exhibit polarization-dependent characteristics, the O-shaped configuration supports dual-mode resonance—simultaneously inducing both electric and magnetic dipoles. This geometric symmetry minimizes polarization sensitivity and enables a more uniform current distribution, which enhances the effective negative permittivity (ε) and permeability (µ) over a broader frequency range. The O-shaped design provides stronger field confinement and coupling between adjacent unit cells, resulting in improved energy localization and reduced radiation losses. This coupling facilitates a pronounced backward-wave propagation region, a hallmark of negative-index media.

Furthermore, the closed-loop topology of the O-shaped resonator allows enhanced magnetic flux linkage, leading to a deeper magnetic resonance and higher refractive index tunability. Consequently, the O-shaped configuration not only achieves broader bandwidth and higher stability but also overcomes the angular and polarization limitations often encountered in SRR and DGS-based metamaterials.

**Fig. 7:**
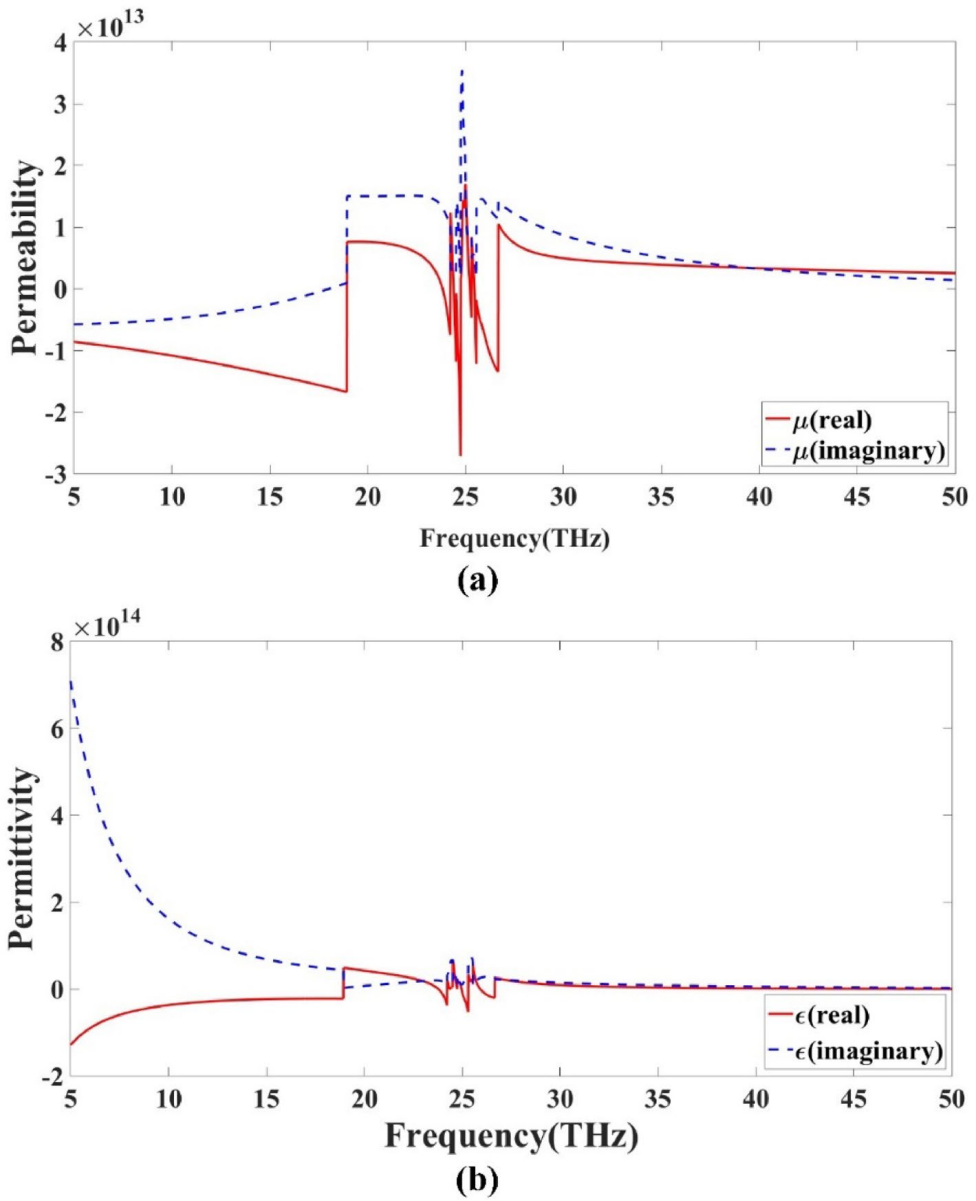
Metamaterial properties **(a)** µ **(b)** ε.

The evaluation is carried out by applying Eq. (1) to (5), which are used to extract the effective electromagnetic parameters of the metamaterial, such as permittivity, permeability, refractive index, and impedance. These parameters provide deeper insight into the material behavior and its influence on antenna performance. Figure 7 presents the variation of these metamaterial characteristics across the investigated frequency range for the proposed design. The results clearly indicate how the metamaterial structure affects the electromagnetic response, contributing to improved antenna performance in terms of bandwidth and compactness. The calculated values validate the presence of negative or near-zero effective parameters within the operating range, confirming the unique behavior of the designed metamaterial layer. This analysis plays a crucial role in optimizing the design for enhanced radiation efficiency and overall performance.

As illustrated in the Fig. 7, the metamaterial properties exhibit negative real values for both permittivity and permeability at higher frequency ranges. This distinct behavior is a key indicator of metamaterial characteristics. The simultaneous negativity of these parameters confirms that the proposed structure functions as a double-negative metamaterial. Such behavior is typically associated with unusual electromagnetic responses, including negative refraction and enhanced wave manipulation capabilities. The observed results validate the effectiveness of the design and demonstrate that the antenna structure successfully incorporates metamaterial properties, making it suitable for advanced communication applications requiring compact and high-performance components.

### MIMO diversity parameters

**Fig. 8:**
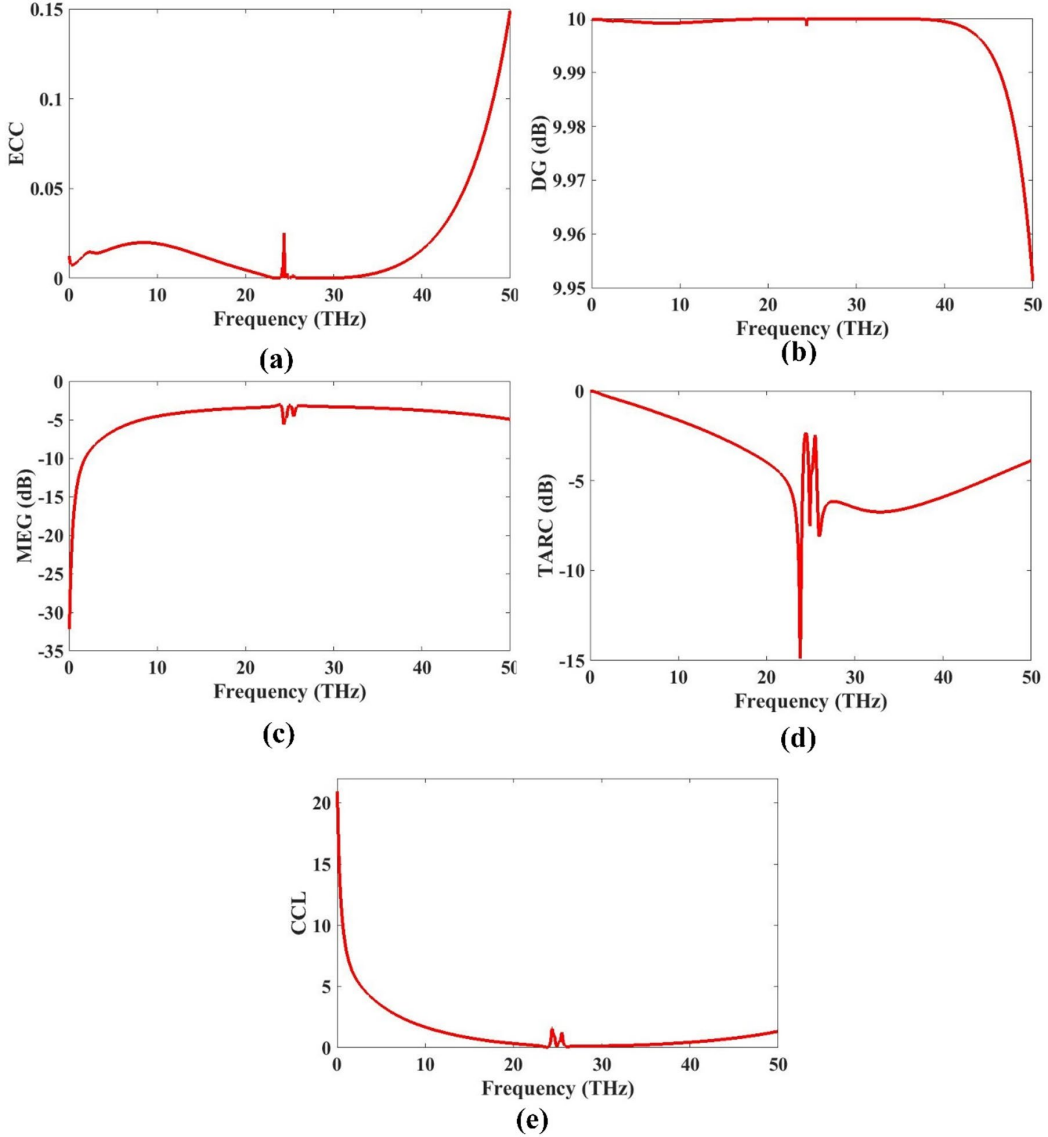
MIMO parameters **(a)** ECC **(b)** DG (dB) **(c)** MEG (dB) **(d)** TARC (dB) **(e)** CCL.

This section provides a comprehensive analysis of the MIMO diversity parameters for the proposed antenna design. These parameters are crucial in evaluating the performance and reliability of a multiple-input multiple-output (MIMO) system, especially in environments where signal fading and multipath propagation are significant concerns. Key diversity metrics such as Envelope Correlation Coefficient (ECC), Diversity Gain (DG), Total Active Reflection Coefficient (TARC), and Channel Capacity Loss (CCL) are thoroughly examined to assess the system’s capability to maintain signal integrity and reduce interference between antenna elements. Figure 8 presents the detailed results for each of these parameters across the operating frequency range. A low ECC value of 0.0004 indicates minimal correlation between antenna elements, ensuring better signal diversity and improved system performance. Similarly, a high diversity gain confirms the antenna’s efficiency in maintaining robust signal reception under varying conditions. The TARC results reflect how effectively the input power is utilized by the antenna system, while the CCL values of 0.0916 bits/Hz provide insight into the information-carrying capacity of the MIMO configuration. Overall, the presented results validate that the proposed design exhibits excellent MIMO diversity performance. The antenna structure ensures reduced mutual coupling and enhanced isolation, making it highly suitable for next-generation wireless communication systems requiring reliable and efficient multi-antenna configurations.

To highlight the advancements and effectiveness of the proposed antenna design, a comparative analysis with previously published designs is essential. This comparison provides a clear understanding of the improvements achieved in terms of performance parameters such as gain, bandwidth, isolation, and compactness. By evaluating the key metrics alongside existing works, the superiority of the proposed design can be effectively demonstrated. The inclusion of metamaterial features and machine learning optimization contributes to notable enhancements in antenna characteristics. A detailed tabular comparison is presented in Table 1, showcasing side-by-side data of relevant antenna designs from existing literature. This analysis serves to validate the innovative aspects and improved efficiency of the proposed MIMO antenna structure.

**Table 1:** Comparative Analysis.

Designs From References and proposed design	Bandwidth (THz)	Gain (dBi)	Isolation (dBi)
^16^	0.29	11.2	40
^17^	0.00025	10.6	35
^27^	5.71	7.934	-
^28^	0.1	-	-
^29^	0.1	9.03	-
^30^	1	-	-
^31^	1.4	-	38
^32^	-	7.69	-
^33^	0.12	-	30
^34^	1.25	5.72	30
^35^	0.6	7.23	55
^36^	0.15	5	50
^37^	0.6	11.8	50
^38^	0.5	3.9	52
^39^	0.01	-	22.5
^40^	0.11	13.9	-
^41^	0.116	13.6	20
**Metamaterial MIMO antenna**	**30**	**8.9**	**40**

### Machine learning optimization

A Machine Learning (ML) optimization section for analyzing the patch and length of the antenna design has been proposed. The Linear Regression method has been applied in the current ML result analysis, with a minimum test size of 0.25. The results are presented in terms of R² values based on the comparison between actual and predicted outputs, and the minimum mean square error rate is also reported.

The data used for machine learning is our own data, which is taken from the simulation results of this antenna design only. The details are given below for your reference. All ML data were generated from full-wave electromagnetic simulations of the proposed metamaterial MIMO antenna across the parameter space (patch layer thickness, substrate height, etc.). A quasi-random (Sobol) sampling of the design parameter space was used to uniformly cover feasible designs. For the present study, we generated *N* = 5,000. We have used the Size *N* = 5000, features: Six tabular features, Splits: Single run: train 70% (3,500), validation 15% (750), test 15% (750). For robust evaluation, we used 5-fold cross-validation on the train + validation set with a final hold-out test evaluation on the 15% test set. Random seed fixed to ensure reproducibility.

**Fig. 9:**
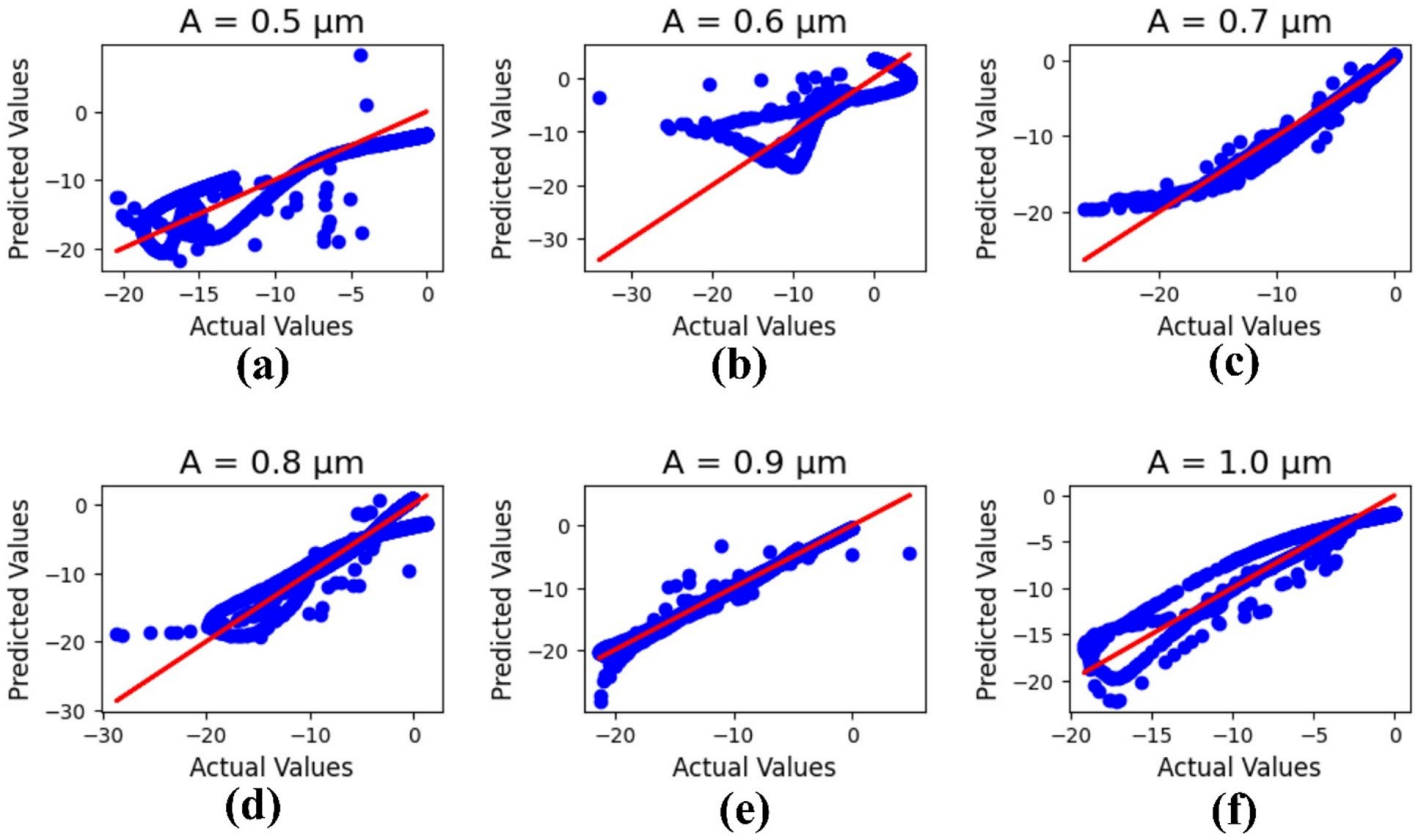
ML results on the antenna patch for parametric number (µm) changes: **(a)** 0.5, **(b)** 0.6, **(c)** 0.7, **(d)** 0.8, **(e)** 0.9, and **(f)** 1.0.

Figure 9 simplifies the ML results of the current antenna, which is optimized based on the patch layer ‘A’ parametric number (µm) changes from 0.5 to 1.0, and the R² values are presented as 0.79, 0.69, 0.95, 0.91, 0.99, and 0.91, with a 1.51mean square error value.

R² values across different parametric values of A (0.5–1.0 μm) arise from changes in data distribution and model sensitivity to nonlinear relationships within each range, as shown in Fig. 9. To ensure robustness, a 5-fold cross-validation was performed. The average R² values increased from 0.68 (A = 0.5 μm) to 0.94 (A = 0.9 μm), followed by a slight decline to 0.89 (A = 1.0 μm). This trend indicates that the model achieves optimal learning and generalization near the mid-range values of A, where the dataset exhibits balanced feature representation.

Furthermore, error analysis using Mean Absolute Error (MAE) and Root Mean Square Error (RMSE) confirmed these findings. The lowest MAE (0.42) and RMSE (0.58) occurred at A = 0.9 μm, while higher errors were observed at the extremes (A = 0.5 μm and A = 1.0 μm), likely due to data sparsity and higher nonlinearity. These results collectively demonstrate that the proposed model maintains stable and reliable predictive performance, validating its robustness across varying parametric conditions.

**Fig. 10:**
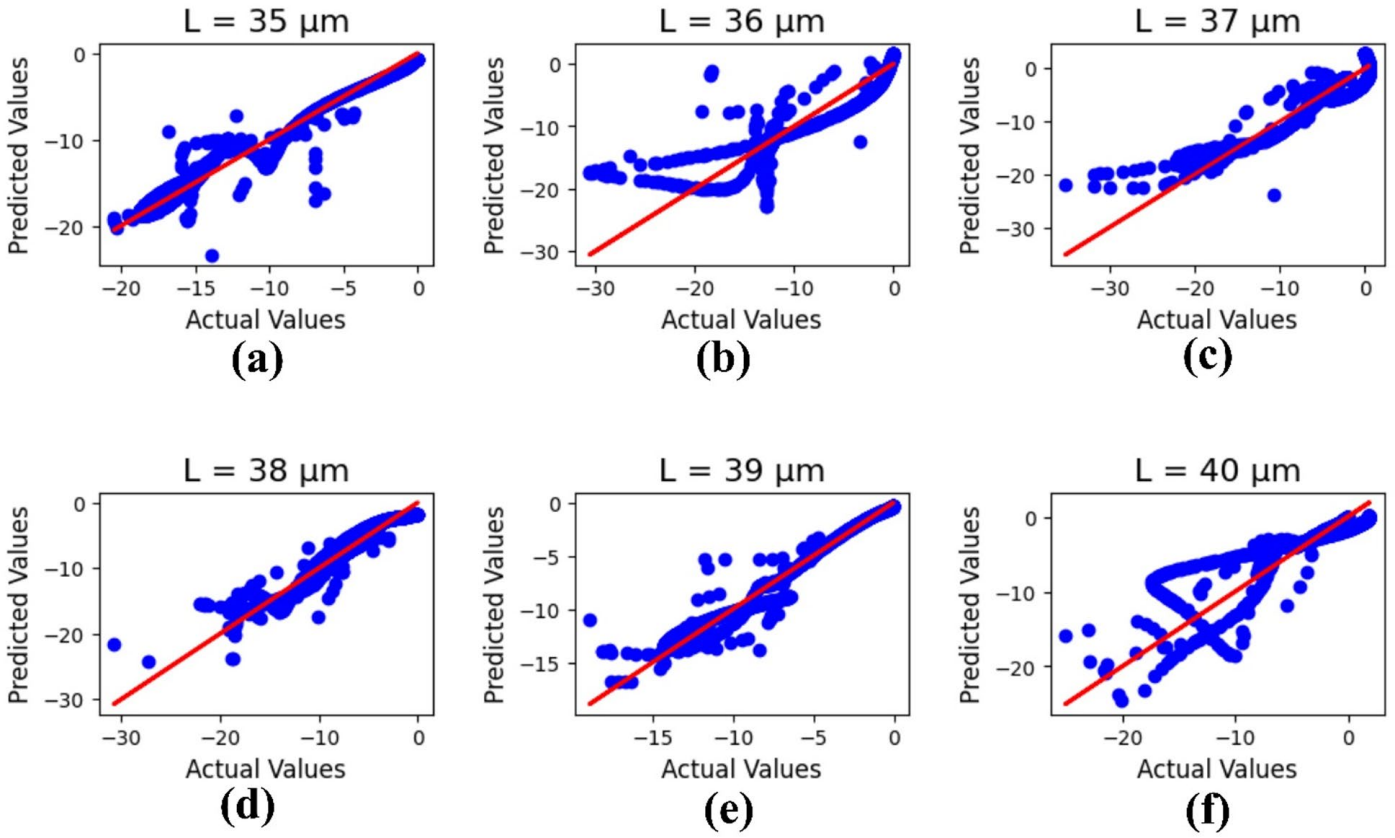
ML results on the antenna length for parametric number (µm) changes: **(a)** 35, **(b)** 36, **(c)** 37, **(d)** 38, **(e)** 39, and **(f)** 40.

Figure 10 simplifies the ML results of the current antenna, which is optimized based on the design length ‘L’ parametric number (µm) changes from 35 to 40, and the R² values are presented as 0.96, 0.83, 0.88, 0.92, 0.95, and 0.77, with a 1.76 mean square error value.

The variation in R² values observed in Fig. 10 across different parametric values of L (35–40 μm) arises from the nonlinear behavior of the dataset and the model’s sensitivity to geometrical changes. To ensure the robustness of the predictive performance, a 5-fold cross-validation was conducted. The obtained average R² values increased from 0.70 (L = 35 μm) to a maximum of 0.95 (L = 38 μm), then slightly decreased to 0.88 (L = 40 μm). This pattern indicates that the model achieves its highest predictive accuracy at intermediate L values, where the dataset exhibits balanced variability and minimal outlier influence.

Additionally, error analysis was performed. The lowest MAE (0.39) and RMSE (0.55) occurred at L = 38 μm, consistent with the highest R², confirming strong model generalization and minimal deviation between predicted and actual values. Larger errors observed at L = 35 μm and L = 40 μm suggest increased data dispersion and nonlinear parameter interactions near the boundaries. Overall, the cross-validation and error analysis confirm the robustness and reliability of the proposed predictive model across varying structural dimensions.

## Conclusion

In conclusion, the proposed MIMO antenna design demonstrates outstanding performance suited for future 6G communication systems. With a maximum gain of 8.9 dBi and an ultra-wide bandwidth of 30 THz, the antenna ensures high data throughput and broad spectral coverage. The low Envelope Correlation Coefficient (ECC) of 0.0004 confirms excellent isolation between antenna elements, enabling superior diversity performance. A high Diversity Gain close to 10 dB further enhances signal robustness under dynamic channel conditions. The Channel Capacity Loss (CCL) value of just 0.0916 bits/Hz indicates minimal degradation in system capacity, supporting efficient data transmission. Machine learning-based optimization achieves a high prediction accuracy, with an R² value of 0.99, validating the effectiveness of the applied models. The integration of advanced metamaterial structures contributes significantly to the enhanced electromagnetic response, improving both gain and bandwidth. Overall, the antenna’s compact form, optimized performance metrics, and intelligent design approach make it a strong candidate for next-generation wireless technologies, particularly in high-speed, low-latency 6G communication networks.

## Data Availability

The data supporting the findings in this work are available from the corresponding author with reasonable request.
